# Waste Electrical and Electronic Fund Policy: Current Status and Evaluation of Implementation in China

**DOI:** 10.3390/ijerph182412945

**Published:** 2021-12-08

**Authors:** Xiao-Shan Yang, Xiao-Xue Zheng, Tian-Yu Zhang, Ying Du, Fengru Long

**Affiliations:** 1 Newhuadu Business School, Minjiang University, No. 200 Xiyuangong Road, Shangjie Town, Minhou County, Fuzhou 350108, China; yangxiaoshan@nbs.edu.cn; 2 The York Management School, University of York, Heslington, York YO10 5DD, UK; 3 School of Business Administration, South China University of Technology, 381 Wushan Road, Tianhe District, Guangzhou 510641, China; 201921034737@mail.scut.edu.cn; 4 The College of Economics and Business Administration, Chongqing Jiaotong University, Xuefu Avenue No. 66, Nan’an District, Chongqing 400074, China; 622200910033@mails.cqjtu.edu.cn

**Keywords:** waste electrical and electronic equipment (WEEE), fund operation mode, life cycle assessment method, EPR, internet

## Abstract

With the accelerated iteration of global electronic and electrical product updates, the demand for electronic and electrical products presents a new trend in which the life cycle of electronic and electrical products is shortened. Waste electrical and electronic equipment (WEEE) products pose a great threat to the global ecological environment, and solving this problem is urgent. Therefore, governments around the world have formulated funding policies for WEEE products, which has led to continuous improvements in such policies. Along these lines, we adopt the circular economy concept, extended producer responsibility theory and life cycle assessment method to comparatively analyse and compare the different fund operation modes in China, Germany, Japan and The Netherlands. In addition, based on the data related to fund policy implementation, we point out the problems in the development of the WEEE industry in China. The analysis results show that although China is the largest WEEE market, it is still in the initial stage and lags behind Western countries in efficiency and cost management. Then, taking as an example ‘Go Green’, an O2O classified recycling platform launched in 2005, this paper performs an extended analysis of the “Internet +” recycling model, which was proposed as a WEEE fund operation solution in China. Finally, we discuss the economic impact of this study on the future implementation and valuation of WEEE fund policy.

## 1. Introduction

With the rapid development of global science and technology, the progress of information and communication technology and the increasing demands of consumers, global electrical and electronic equipment (EEE) is being updated with a faster iteration speed and shorter life cycle. A large amount of waste electrical and electronic equipment (WEEE) has been generated worldwide, and it accounts for 8% of all municipal waste [1]. WEEE is a hazardous waste if it is not handled properly [2] and can pollute soil and groundwater with harmful substances, such as lead-based solder, arsenic and selenium, thus posing a threat to human health and the environment [3]. In terms of the environment, it is imperative to recycle reusable parts and basic materials of WEEE products, especially copper and precious metals. In terms of econometrics, the recovery rate from one ton of mobile phones is equivalent to 40 times the recovery rate from one ton of gold ore, and the value far exceeds the value of ore. If WEEE products are appropriately treated, social and economic benefits will be realized with WEEE recycling. Therefore, many countries have established collecting and recycling systems and developed relevant policies regarding WEEE recycling [4].

As the largest manufacturer and the second largest commodity-consuming country, China produced 7.2 million tons of WEEE in 2016, which is the highest in the world [5]; however, only approximately 3.66 million tons of WEEE were recycled [6]. As the world’s largest producer of EEE and importer of WEEE, China’s reprocessing of imported cheap solid waste can drive the employment and GDP growth of some coastal cities and the development of some secondary markets [5]. The cost generated from reprocessing waste is considered an ecological cost and will inevitably occur in the future [7].

Approximately 4000 tons of WEEE are estimated to be exported globally every hour, and 80% of this waste is exported to Asia. Among the waste exported to Asia, China accounted for 90%. Guiyu in Guangdong Province and Taizhou in Zhejiang Province are two major exporting destinations [8]. After WEEE arrives in China, it is recycled through informal recycling channels and processed through simple and crude non-standard recycling and dismantling, which has caused great harm to the environment and posed a great threat to human health [9]. Therefore, China is constantly facing serious e-waste problems caused by increasing domestic power generation and foreign imports [10]. To solve this problem, the Chinese government promulgated the Regulations on WEEE Recycling Management in 2009 and the WEEE recycling tax and WEEE fund policy in 2012. According to the funding policy, producers and importers pay for disposal costs while qualified dismantlers and disposers receive subsidies under the distribution and supervision of the government [3]. Previous research and practice show that this promulgated funding policy is very important for guiding improvements to relevant national fund policies [11].

Due to the positive influence of government policies, the global WEEE recycling industry has developed rapidly. In an ideal state, the realization of a circular economy depends on the utilization of “urban mines”, which refer to a large number of valuable resources, such as rare metals in electronic and electrical products in urban areas [12]. Also, based on the previous literature, in WEEE management, the extended producer responsibility (EPR) is believed to be essential for the enforcement of circular economy [13] EPR programme usually concerned with the establishment of a recycling plan, according to which consumers return used products to producers and the latter should take the responsibility for the reuse, refurbishment, remanufacturing and recycling of such products. In this case, the cost of WEEE management is internalized to the producers and therefore, transit the burden of WEEE management from consumers to producers [14].

However, under the temptation of huge profits, many problems will inevitably occur in the development of the industry. In China, high-cost methods of disposing of waste electronic products have been used for a long time because the harm and recycling value of electronic wastes is not properly understood by the public, which is primarily driven by a lack of consideration of safety and environmental factors and economic returns. Over time, illegal dismantling has formed a covert informal supply chain that has restricted the available space for formal and standard dismantling enterprises [2].

As the dismantling fund helps normalize and legitimize the industrial chain, it has become an important source of income for legal dismantling enterprises at the present stage. However, if subsidy allocations are not in place, then formal and standard recycling enterprises will have to reduce the scale of dismantling and electronic waste will once again flow into illegal dismantling channels. However, the longstanding problem associated with the low income of the dismantling fund is due to the insufficient fund recovery capacity [11].

The data show that the recycling amount of WEEE products has increased from 114 million tons in 2013 to 164 million tons in 2017; however, the actual income of the dismantling fund has hovered between 2.6 billion and 2.8 billion RMB in the same period. Except in 2013 [15], the actual collection of dismantling funds was unable to meet the income budget. As a result, the dismantling fund falls into the dilemma that it must release funds to dismantling enterprises in the case of insufficient capital.

In 2017, WEEE subsidies almost stagnated, and subsidies for dismantling funds were not paid on time [16,17]. Therefore, it is difficult for dismantling enterprises to obtain funds through subsidies to support their cash flow and daily operations. In this case, subsidized formal enterprises have to reduce the scale of their dismantling.

The heavily burdened dismantling fund has attempted to promote the development of the e-waste recycling industry; however, it is caught in a dilemma. If the fund continues to offer subsidies, then it needs to overcome a huge deficit. If the fund is stopped, formal enterprises will lose an essential source of financial support, which will encourage the rampant illegal dismantling operations in the industry. Compared with China, foreign countries have not only established sound fund management systems but also enacted supporting laws, regulations and recycling standards to ensure and standardize the smooth implementation of the fund system [1].

According to national and regional conditions, actual needs and economic development levels, each country and region has formulated corresponding catalogues for WEEE. For example, in the management of its catalogue of WEEEs, the United States pays more attention to electronic products with display screens, such as televisions and computer monitors. European countries, however, include almost all kinds of electronic products in their catalogues [1]. The formulation of catalogues in Japan and South Korea usually focuses on electronic products with a high market penetration rate and large resources. In conclusion, foreign waste recycling standards cover almost the whole industrial chain, such as producers, consumers and dismantling enterprises, while China’s standards are more about standardizing dismantling enterprises. Therefore, for China, it is necessary to learn from the fund policy experience of developed countries, further improve the extended producer responsibility system, optimize the fund collection and subsidy mechanism, and adopt a series of supporting policies to realize healthy development of the industry and promote the development of circular economy in the whole society.

To achieve the primary objectives, this study aims to answer the following questions:

(1) What is the operation mode of the WEEE fund policy in China?
(2) How does fund policy implementation and the WEEE industry change in China?
(3) What are the main problems and solutions for the WEEE fund policy in China?

## 2. WEEE Fund Operation Mode in Different Countries

The iteration of EEEs is accelerated by continuous technology innovation and market demand expansion. In this case, the service life of electronic and electrical products is greatly shortened, which results in a large amount of WEEE. Considering the serious environmental problems created by WEEE and the high use value of the associated materials, such as metals, precious metals, plastics and glass, the recycling treatment of WEEE has become a hot spot. Many countries’ political decision-makers have reviewed the existing policy options and drawn the conclusion that extending the responsibility of producers to the end of the product life cycle can alleviate the environmental pressure generated by these waste streams [18]. Some developed countries have formed more advanced and reasonable WEEE recovery and charge systems that vary according to national conditions. There are a number of studies have explored the WEEE management system and fund operation mode, especially in developed countries [19,20]. In the last two years, there has been a growing number of research works on WEEE management in China, however, only a few studies compare WEEE management between China and other developed counties [1].

This paper introduces the most typical WEEE system in developed countries, including China, Germany, The Netherlands and Japan. Then, it summarizes the responsibility system and payment mode of WEEE in developed countries.

### 2.1. Germany

As a waste management measurement, extended producer responsibility was first introduced in Germany in 1991 [21]. As the first country to implement extended producer responsibility, Germany shows relatively mature performance in terms of the global management of solid waste treatment [22]. In Germany, the WEEE fund collection and subsidy system stipulates the specific obligations of all relevant stakeholders (manufacturers, trade, municipal authorities, owners and disposers) in the management of e-waste [23]. According to the principle of extended producer responsibility, after the EEE produced by an enterprise is scrapped, the manufacturer and importer are responsible for managing the scrap [24], collecting the corresponding amount, and paying the corresponding subsidy to the processor.

**Figure 1:**
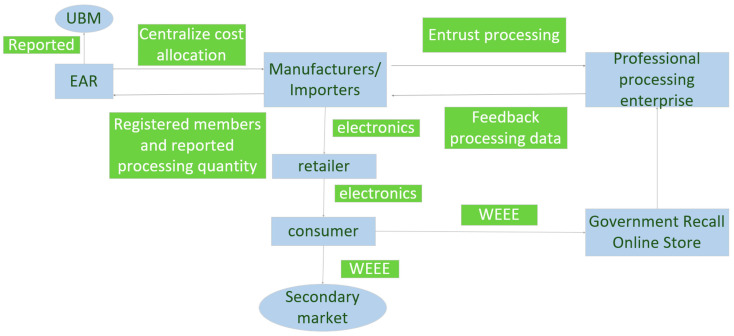
Operation mode of the fund policy in Germany (concluded from [22,25]).

Every producer must provide guarantees when putting products on the market to ensure that the collection, treatment and disposal of products are funded [10]. The whole process is supervised by the German EAR Foundation [23]. The main work includes counting and checking the number of electronic products sold by importers and manufacturers, calculating the number of subsidies allocated to processing enterprises according to the processing difficulty and quantity, and collecting the amount of funds according to this ratio. The specific operation mode is shown in Figure 1.

The figure shows the fund operation mode of the German system. First, producers should register as members in the special management organization, namely, the EAR Foundation, and provide the required information. In return, the EAR only sends a registration number to each registered manufacturer, and the producer must use this number in all commercial transactions. In practice, German manufacturers usually do not directly participate in the transfer, transportation, treatment and disposal of WEEE but entrust processing companies to carry out specific operations. After the used products are disposed of by distributors and retailers, some reusable products can flow into the secondary market [10] for renovation and resale after being tested and sorted by the dismantling company. As far as the WEEE collection system is concerned, consumers of non-recyclable EEE can send WEEE to more than 1400 collection points established by local authorities [19,26]. Moreover, professional processing enterprises should give feedback on the processing data to manufacturers. WEEE’s government recycling network is handed over to professional WEEE processing enterprises for dismantling and disposal. Factories that deal with e-waste may produce emissions and noise. Therefore, according to the Emission Control Act in Germany, the establishment of waste treatment plants requires government approval and the number of emissions monitored and controlled by competent authorities is strictly limited.

Professional processing enterprises are entrusted by product manufacturers and in a completely competitive relationship with manufacturers. Processing enterprises compete with manufacturers’ processing orders according to their scale, technology and labour conditions and return the data generated by the recycling processing results to manufacturers. Some production enterprises have set up their own recycling departments, and their waste products are directly returned to the production enterprises through their own or other channels. Then, the manufacturer uniformly reports the recycling capacity to the EAR Foundation (including those handled by the manufacturer itself and those entrusted by professional processing enterprises). The EAR Foundation performs calculations according to the manufacturer’s sales volume and total processing volume and reports the subsidy amount for the processing enterprises to the environment agency (UBA) according to the standard.

Professional processing enterprises in Germany also need the government to certify their processing qualifications or prove their processing technical ability in voluntary certification activities to help manufacturers handle orders and win customers’ trust. To date, there are approximately 700 processing enterprises in Germany. However, according to the EU enterprise classification standard for the number of enterprises and annual income, the processing enterprises are mostly micro-, small- and medium-sized. Moreover, an optimal competition mode between processing enterprises can promote long-term strategic cooperation between manufacturers and processing enterprises, such as by encouraging the sharing of product information among them, enabling manufacturers to collect more reliable and complete data and information in product design, improving product characteristics, and reducing recovery costs and price games [27]. On the other hand, competition only allows enterprises with lower recycling and processing costs to occupy a dominant position in the market and promote the development of the industry.

The EAR Foundation only supervises manufacturers and importers, who share the responsibility of recycling. However, in the recycling process, the foundation did not distinguish the brands and categories of waste products and raw materials and could not distinguish the responsibilities of different manufacturers. Additionally, the foundation cannot recognize the hidden costs of green manufacturing. In this case, some opportunistic enterprises will have the chance to increase their recycling and processing and thereby reduce other enterprises’ profits.

### 2.2. Japan

Due to the limitation of Japan’s own resources and environment, the rapid development of electronic information technology and the economy, and the popularization of electronic products, the corresponding number of WEEE is increasing. Since the end of the last century, unsustainable development has caused considerable waste, polluted the environment, endangered people’s health and hindered sustainable development.

Therefore, the Japanese government has taken the lead in transforming the original traditional economic model into a circular economy to improve the utilization rate of resources. Since 1970, the Japanese government has implemented a series of measures to conserve resources and develop energy-saving products. In 2000, Japan promulgated the Basic Law for Promoting the Establishment of a Circular Society and then a series of related laws, which gradually transformed Japan from a “public nuisance” country to an “advanced country in environmental protection” [28]. In developed countries, the legislation of the circular economy is becoming mature.

**Figure 2:**
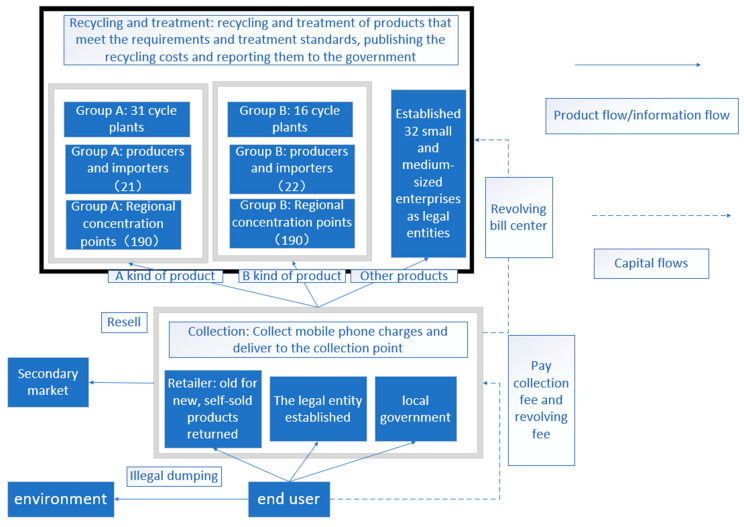
Operation mode of the fund policy in Japan (concluded from [30,31]).

In Asia, Japan took the lead in formulating WEEE policy. In 1998, the Household Appliances Recycling Law came into effect, and it stipulated that consumers should bear WEEE recycling costs. Subsequently, Japan launched its own WEEE recycling system in 2001, and approximately half of the electrical appliances used were disposed of after collection [29]. The operation mode of Japan’s WEEE fund policy is shown in Figure 2.

The figure shows that the WEEE management system in Japan is based on “fee withdrawal” [32]. When discarding wasted products, consumers handle the WEEE to specific collection depots. These depots are designated by either WEEE retailers, legal entities or local governments. The cost of WEEE collection and recycling is borne by consumers. Then, third-level manufacturers will receive the collected WEEE and the amount of capital charged from consumers for WEEE recycling. Japanese municipal authorities do not carry out their own recycling and disposal activities like some other countries. The collection fees and funds are managed by the Household Electrical Appliances Association, while the collection and processing are carried out by Group A (Panasonic = Toshiba Group) and Group B (Mitsubishi = Hitachi Group) under the cooperation system of manufacturers [29]. WEEE is classified by the second-level manufacturers according to the product brands of groups A and B and distributed to different manufacturers’ alliances, and the manufacturers concentrate on WEEE recycling.

A significant feature of the operation of Japan’s WEEE fund is that there is no special foundation or government agency to manage the recycling of WEEE in a unified way, although consumers and manufacturers take full responsibility, which reduces the fund management cost and shortens the capital turnover cycle [18]. In addition, information on the whole return process of products from consumers to manufacturers is recorded by the revolving bill centre. An advantage of this system is that it can track the flow direction of products according to information and promote the standardized disposal of discarded products.

### 2.3. The Netherlands

As a member of the European Union, The Netherlands passed the WEEE Recycling and Utilization Law in 1998 and passed the E-Waste Management Law in 2004, which covered all types of e-waste specified in the WEEE directive of the European Union. During this period, challenges associated with the application of WEEE directives in EPR systems have been observed.

**Figure 3:**
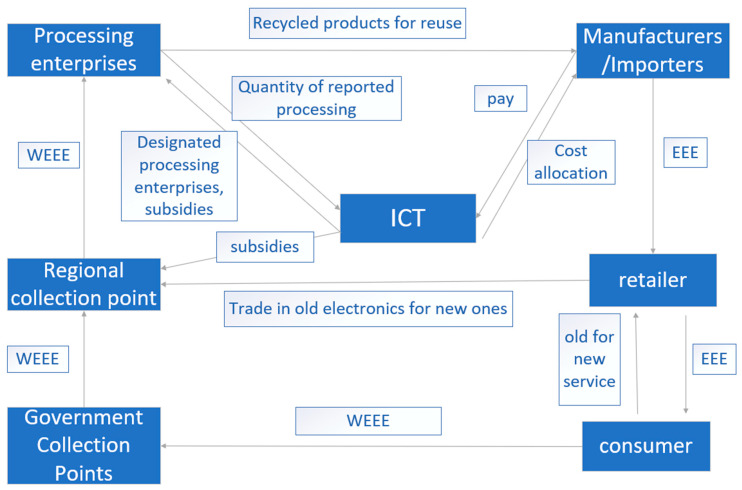
Operation model of the Dutch information technology and telecommunications (ICT) waste electrical and electronic equipment (WEEE) fund system (concluded from [18,33]).

The country seems to be relatively successful. In 2014, it was reported that 44% of the e-waste (320,000 tons) on the Dutch market was collected and disposed of [18]. Compared with Japan and China, The Netherlands has no professional organization to supervise and regulate WEEEs. The ICT (IT and telecommunications) Environmental System and the Nederlandse Verwijdering Metalelektro Producten (NVMP) have mainly been established to supervise and regulate WEEE products. The two funds are responsible for different groups of products, with the former mainly responsible for IT equipment, photocopiers, printers and other products and the latter mainly responsible for the sales volume and cost accounting of television, refrigerators and other products. The operation mode of the ICT is described as shown in Figure 3.

The figure shows that retailers are obliged to recover WEEE from Dutch consumers, who can take WEEE to retailers for trade-in or to government collection points. The retailers then trade the old WEEE, and the government collects the WEEE directly and transports it to regional sorting stations [13]. Then, the WEEE flows to the processing enterprises, which sell the recovered products directly to producers and importers and report to the ICT system. The ICT then designates WEEE recycling enterprises and regional collection points and processing enterprises for subsidies. Processing enterprises are designated by the ICT system to avoid market competition. The ICT system processes the quantity and weight of waste products as well as the sales price and quantity of waste products fed back by the processing enterprises. After accounting, the costs are allocated to determine the number of subsidies.

In summary, one of the advantages of the Dutch fund policy model is that because the ICT system stipulates the recycling processors, the manufacturers and processors can establish a long-term and stable cooperative relationship, which is conducive to maintaining the long-term stability of the recycling system [34]. By supervising electrical products by the two foundations, the proportion of WEEE recycling can be improved, the recycling effect is better, the binding force on enterprises is greater, and the adverse impact on the environment will naturally be minimized. However, the operation of foundations requires considerable costs and enterprises cannot easily achieve low-cost efficiency.

### 2.4. China

As the world’s largest EEE producer and WEEE importer, China has its unique social system and policy environment, which makes the WEEE fund policy hard to directly transplant to China’s WEEE market. Also, the WEEE management system is less complete than that of the developing countries, and therefore the treatment method of WEEE is China is more irregular than other countries. In this case, Chinese government are engaged to find out and implement policies that suitable for China’s current status of WEEE market [35].

China has experienced four stages in the development of WEEE recycling. The first stage was the traditional recycling mode of renewable resources under the market economy system before 2009. In the second stage (2009–2011), the major participants included retailers and manufacturers and the recycling mode was trade-in policy and governmental subsidies. In the third stage (2012–2015), the recycling mode under the regulations and fund system is the traditional recycling mode of renewable resources based on individual recycling. The fourth stage is considered the development stage (after 2016), in which the traditional recycling mode and innovative recycling mode coexist. In the fourth stage, however, due to the early emergence of informal dismantling enterprises in the market, environmental costs are not considered. Therefore, the profits are much higher than that of formal dismantling enterprises [36]. Consequently, the amount of WEEEs recycled by formal dismantling enterprises is limited. To solve this issue, China began to implement a stricter qualification system for WEEE recycling enterprises and gave financial subsidies to high-quality recycling enterprises.

This paper mainly studies the pollution control of WEEE recycling in China. In 2005, the Law on Prevention and Control of Environmental Pollution by Solid Waste was promulgated, which stipulated the responsibilities of producers, retailers, importers and consumers. Meanwhile, the government encourages companies to make a transition towards circular economy for the sustainable development of society [37].

Therefore, China draws on foreign advanced WEEE fund operation modes and introduces the original legislation system on EPR recovery so that manufacturers are responsible for the environmental impact of the whole life cycle of products, including the consumption process of products as well as the design of products, which can be comprehensively considered until the products are discarded. As of 2021, 109 Chinese enterprises have obtained formal processing qualifications. However, China’s EPR system is still in its infancy. When establishing the operation mode of WEEE funds, we should consider the current situation of China’s EEE industry and the basic situation of the recycling and dismantling market.

First, for the EEE industry, due to the influence of supply and demand in recent years, a large number of manufacturers have entered the electronic product market, resulting in oversupply. Thus, the electronic product market is a buyer’s market, where sellers compete with sellers and prices are low. For example, the average industrial profit rate of China’s electronic product manufacturing industry in 2014 was only 5.2%, which was lower than the average level of 6.5% in all industries [38]. Due to oversupply, consumer demand is not strong enough and consumers are in a dominant position in the market, which makes it difficult to transfer WEEE treatment funds from producers to consumers. As a result, the cost of capital is difficult to pass on to consumers.

Second, China’s WEEE recycling process is characterized by both formal and informal recycling. Informal recycling is widespread in China. Waste recycling requires no special skills and has become an important means for informal collectors to make a living [39,40,41]. Most of the waste collectors are vendors, collection stations, distributors and intermediaries. The large amount of labour engaged in waste recycling and the complicated collection process structure make subsidy implementation hard to achieve [11].

Finally, EEE disassembly and utilization are the main sources of environmental impacts when compared with recycling [42]. An important role of WEEE funds is to compensate for the loss of the dismantling company and improve its competitiveness. Since informal recycling and disassembly does not consider environmental costs, its profits and market share are much higher than those of formal recycling enterprises.

To solve the aforementioned problems and standardize the WEEE recycling industry, China has implemented the WEEE disposal fund policy, charging recycling fees from producers to subsidize the formal recycling sector. Specifically, the dismantling of electronic products must be scrutinized by environmental authorities, while dismantling certificates should be issued by formal recycling departments [43].

**Figure 4:**
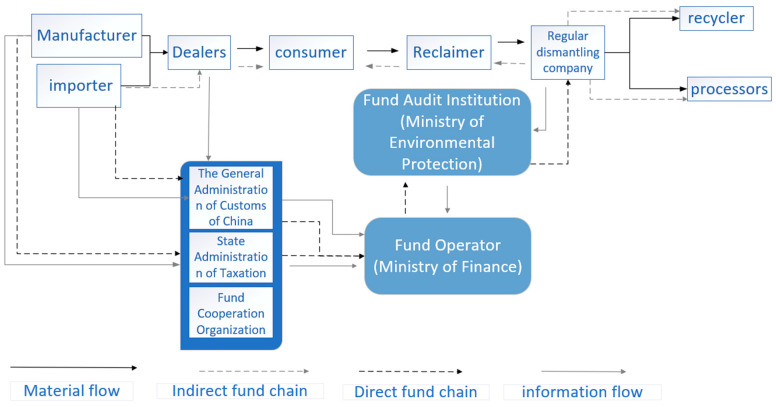
Operation mode of the fund policy in China (concluded from [44]).

In addition, the Ministry of Finance collects EEE sales invoices to determine the sales of manufacturers and importers. Under the supervision of the Ministry of Finance, the State Taxation Administration and Customs are responsible for collecting the production and consumption of domestic EEE and imported EEE, respectively, and transferring them to the disposal fund. Finally, the state-certified formal processing enterprises submit WEEE recycling volume reports to the Ministry of Environmental Protection every year, and the Ministry of Environmental Protection provides subsidies from the fund pool after examination. The examination period of the Ministry of Environmental Protection is usually 6–12 months, which means that formal processing enterprises can only obtain subsidies at one year after submitting an application. The details are shown in Figure 4.

### 2.5. Comparative Analysis

A comparison of the fund operation characteristics of different countries shows that the operation mechanisms of producer extended responsibility in these four countries are different. The comparative analysis results are presented in Table 1.

First, there are two fund collection modes: producer payment mechanisms (such as Germany, The Netherlands and China) and consumer payment mechanisms (such as Japan). The first mode indicates that the fund is collected in the production stage, which will increase the burden on producers. Although producers can pass some of the burden on to consumers, this will be hard to do if there are many producers and fierce competition. For consumer payment, the fund is collected when the product is scrapped and customers are charged to discard the product. The consumer payment mechanism is less frequently used, although it reduces the generation of WEEE to a certain extent. Without a strict process for capital recycling, capital flows directly from the consumer to processing enterprises.

Second, the participants in the fund’s subsidies include collectors (Germany), dismantling companies (China and Japan), or both (The Netherlands), and they are all regulated and supervised by the government.

Third, capital operation agents can be national (such as Japan and China) or professional (such as Germany and The Netherlands). The advantage of the former is that the operation mechanism of the fund can be considered as a whole by the government and combined with some other national policies, while the advantage of the latter is that it can avoid monopolies and introduce competition mechanisms, which greatly reduce the management costs.

Fourth, the WEEE policy can be managed by the government, such as Germany, The Netherlands and China. Additionally, in some countries, such as Japan, consumers and manufacturers are responsible for this activity. The operation of the WEEE fund policy in China is different from that of Japan. However, the fund policy largely depends on government restrictions. For example, the amount of tax payable and subsidy granted is decided by the state, while the content of the recycling category is enacted by the government. In this case, most enterprises passively undertake social responsibilities. The subsidy distribution cycle is long, and formal recycling enterprises are not active enough, resulting in a low standard recycling rate.

Fifth, WEEE is usually recycled by collection points set up either by the government or producers. Although China has built a certain number of recycling sites, because of the high cost of recycling, immature recycling technology, and deficient consumer recycling environmental protection awareness, a complete system of recycling waste electric and electronic products has not yet been built.

Compared with Japan, the WEEE treatment in China, Germany and The Netherlands is not categorized by brand and type. However, the differentiation of all kinds of waste products before recycling can effectively improve recycling efficiency and motivate manufacturers to consider ecological and environmental impact factors in product design and production, thus promoting green production [45].

Moreover, consumer behaviour will have a great impact on enterprises. Compared with The Netherlands and Japan, in China, the source of disposal cost of WEEE consists of two parts. The first part is collected from producers and importers. The second part is collected from consumers. Consumers have to bear some or even all of the costs of recycling. However, in the operation of fund policies in China, there are no clear laws and regulations to restrain consumer behaviour, resulting in a high rate of informal recycling and disassembly, which has a great impact on the environment.

China’s fund policy implementation lags behind, resulting in many problems in the current policy implementation. Based on the evaluation of foreign WEEE fund policies, this paper will then explore the suitable WEEE fund policy that conforms to the national conditions of China.

## 3. Comprehensive Evaluation of the Effect of China’s Fund Policy Implementation

This section briefly introduces how the numbers of EEEs and WEEEs change in China. This analysis is carried out following the evolution change of the subsidy standard of the China WEEE disposal fund.

### 3.1. Output of Electrical and Electronic Equipment (EEE) in China

**Figure 5:**
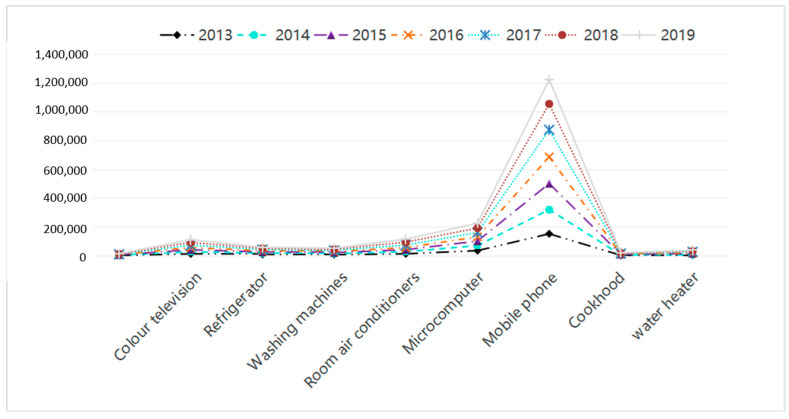
Household output of electrical and electronic equipment (EEE) in China (10,000 units).

The reform and opening-up policy has boosted China’s development in information and communication technology, which makes the consumer electronics industry one of the fastest-growing industries in China. Its rapid growth is reflected in the significant growth of the total number of mobile phone users, which reached 1.605 billion in 2020. In addition, the number of 5G mobile network users reached 310 million in 2020. As the demand side of the EEE industry is gradually saturated, the profit of China’s EEE industry has decreased dramatically, which indicates the urgency of transforming and upgrading the business model in this industry. Mobile phone is a representative EEE in China. The outputs of other EEEs in China from 2013 to 2019 are shown in Figure 5.

**Figure 6:**
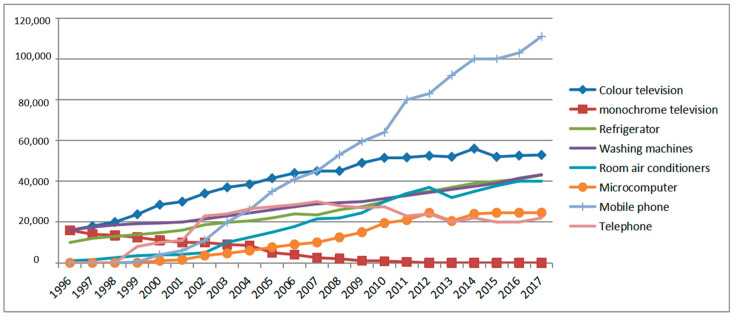
Household output of electrical and electronic products in China (10,000 units).

The changes in household output of EEEs in China from 1996 to 2017 are shown in Figure 6. A continuous increase is observed in the number of new electronic products, including mobile phones, colour television, washing machines, air conditioners and microcomputers.

### 3.2. Challenges of Waste Electrical and Electronic Equipment (WEEE) Disposal in China

The large quantity of EEEs in China lays a basis for the development of the WEEE industry. The large quantity of China’s EEEs lays a foundation for the WEEE industry. because with the faster iteration of EEEs, the large production volume of EEEs indicates the inevitable increment in the numbers of WEEEs. At present, the number of home appliances in China has reached 2.1 billion units. In 2020, 160 million household appliances reached their standard life spans. Faced with such a huge number of products to be discarded, the recycling capacity in China is seriously insufficient. The recycling market is dominated by informal recycling channels. Previous statistics show that only approximately 80 million WEEEs are dismantled annually by the formal recycling channel, indicating that at least half of the potential WEEEs (160 million) will flow into the informal channel in 2020. Recycling of these products to be discarded poses great pressure on China’s WEEE recycling industry. Additionally, formal WEEE recycling and dismantling enterprises are not formal traders. Compared with informal recycling enterprises, formal enterprises do not have price advantages in purchasing WEEEs. Without a price advantage, formal channels are generally not welcomed by consumers.

To standardize WEEE recycling and promote resource utilization and circular economic development, the Chinese government has promulgated a series of industry laws and regulations. For example, in 2009, the State Council promulgated the Regulations on the Management of Recycling of Used Electronic and Electrical Products (hereinafter referred to as the Regulations).

The Regulations first clarify the WEEE catalogue and then establish a series of systems, including disposal funds systems, processing enterprise qualification licensing systems and enterprise information processing systems. Among them, the disposal fund system is an important way of implementing the extended responsibility system for EEE producers in China. Since the implementation of the disposal fund system, the growth trend of the WEEE industry has not been optimistic primarily because of the contradiction between WEEE production capacity and WEEE recycling capacity.

### 3.3. Analysis of Subsidy Standard Adjustments for WEEE Disposal Funds in China

To reasonably guide the recycling and treatment of WEEEs and accelerate the improvement of the technical level and overall efficiency of the industry, China has adjusted the subsidy standards of WEEE disposal funds since 2016 according to the changes in the recycling and treatment costs and benefits of waste electrical and electronic products. This section briefly analyses the changes in the amount of dismantling of major WEEEs in China’s formal processing enterprises and the content and impact of the adjustment of the WEEE fund subsidy standard in China.

To reasonably guide WEEE recycling and accelerate the improvement of the industry technical level and overall efficiency, since 2016, China has adjusted the subsidy standard of WEEE disposal funds according to the change in cost and benefit of WEEE recycling. This section briefly analyses the changes in the numbers of major WEEEs in China’s formal processing enterprises and then analyses the contents and impact factors of the adjustment of subsidy standards for WEEE disposal funds in China.

#### 3.3.1. Changes in the Number of Major WEEEs in China’s Formal Processing Enterprises

The WEEE fund policy has led WEEE recycling enterprises to focus on environmental impacts and resource recycling. However, the illegally dismantling channels in China still account for a large proportion.

**Figure 7:**
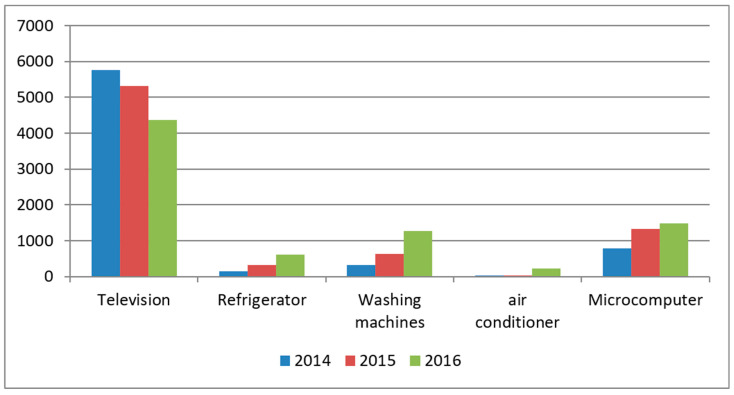
Actual quantity of major WEEEs dismantled in qualified processing enterprises in China from 2014 to 2016.

The major WEEEs in China are televisions, washing machines, refrigerators, air conditioners and microcomputers. The dismantling quantity of these products presents a large difference. Figure 7 shows the dismantling quantity statistics from 2014 to 2016.

In 2016, the actual dismantling amount of major WEEE in China’s formal processing enterprises increased continuously, and the green recycling rate (i.e., referring to the ratio of the actual dismantling amount to the theoretical scrapping amount.) increased from 37.94% in 2013 to 70.77% in 2016. As shown in Figure 7, the quantities of television dismantling are the highest, although a downward trend is observed in television dismantling quantities and an upward trend is observed in the dismantling quantities of the other four EEEs. This phenomenon may result from the substantial adjustment of the subsidy quota of the WEEE fund in 2016.

#### 3.3.2. Analysis of the Contents and Influence of the WEEE Fund Subsidy Standard Adjustment in China

On 26 November 2015, the state issued a document to further adjust the subsidy of the WEEE disposal fund. The main adjustment objects are the aforementioned five major WEEEs. The subsidy standard content is adjusted according to the actual market changes. The new standard was officially implemented on 1 January 2016. In Table 2, the content of the new standard is compared with that of the previous standard.

Table 3 illustrate the scope and standards of the funds levied by China on enterprises selling EEEs. Based on the above data, Table 4 is developed.

Based on the data presented in the tables above, the following points can be directly obtained.

First, there are ups and downs in the adjustment of the fund collection amount. Before 2016, the adjustment number of subsidies for washing machines and air conditioners was the same and the number of subsidies was the same at 35; however, the financial subsidies on air conditioner adjustments increased significantly in 2016. The recycling rate of air conditioners in 2014 was only 0.74%, which represented the lowest proportion. In the same year, the number of recycled televisions accounted for 98.7% of the total recycling amount. Since fund policy implementation began in 2016, the number of major WEEEs flowing into formal processing enterprises has increased from approximately 16.4% to 58.8%. However, television recycling accounted for a large part of the growth, and the recycling quantities of the other four WEEEs in formal channels also showed significant growth.

Second, the new standard excludes televisions under 14 inches from subsidies because a large proportion of those televisions have been recycled and most manufacturers have halted the production of such televisions. Additionally, technological progress in dismantling and processing enterprises has reduced the cost of dismantling small products; therefore, it is unnecessary for the government to subsidize them in small product recycling.

Third, the recycling subsidy of individual EEEs is much lower than that of informal processing enterprises. Producers include in the product sales price their costs, such as product development and sales, and cost removal is not involved in the recycling process. Under the condition that the unit cost of formal processing is not high, informal manufacturers will renovate some products and the market price will be much higher than that of formal processing enterprises.

Fourth, the data show that the government’s standards for all EEE manufacturers’ products have not changed and differences are not observed between the same product type and different product types. Therefore, the government’s behaviour will lead to a certain degree of competitive advantage only for low-cost enterprises in the market, which will encourage enterprises to implement technological and product innovation and strive to achieve the highest value at the lowest cost.

With the adjustments of the contents of the WEEE fund subsidy, enterprises can benefit from setting up their own WEEE recycling channel and department. For example, in China, Gree is one of the large-scale production enterprises and has realized a closed loop in their recycling activities. Gree’s products and their parts can eventually flow back to enterprises. Thus, enterprises can obtain all kinds of information about the whole life cycle of products and maximize the efficiency of product design innovation. Their recycling and dismantling behaviour can enable them to obtain financial subsidies and gain a reputation in the market.

## 4. Problems Associated with China’s Fund Policies

The WEEE recycling industry is an important part of the electrical and electronic industry chain, and it is an important factor in developing a circular economy development mode in the electrical and electronic industry [46]. Additionally, it is the first link for resource reuse, resource savings and energy savings. Therefore, the WEEE recycling industry should not be underestimated. In view of the difficulties of the WEEE recycling industry at present, the relevant government departments have provided considerable policy support to the development of the WEEE recycling industry. The WEEE recycling industry is a new environmental protection industry in China that has developed rapidly and formed a new industrial group.

However, there are several problems in the development of the WEEE recycling industry. Some processing enterprises are in a state of semi-production suspension, some WEEE recycling enterprises are loss-making, and some WEEE recycling enterprises have closed down. There is also fierce competition in the WEEE recycling industry. Identifying a method of promoting the normal and healthy development of the industry as soon as possible is worth discussing. This section attempts to analyse the greater development dilemma faced by formal e-waste dismantling enterprises in China from several aspects.

### 4.1. Operational Issues for Dismantling Enterprises

The formal dismantling enterprise has stagnant growth, and its overall operating rate is insufficient.

At present, China has enacted an industry access policy for waste electrical appliances and electronic processing industries. The company must obtain the technical and infrastructure standards after WEEE dismantling qualification, and then the government should include the enterprise in the WEEE recycling and disposal fund subsidy list so that the enterprise can apply for fund subsidy to meet the dismantling amount.

**Figure 8:**
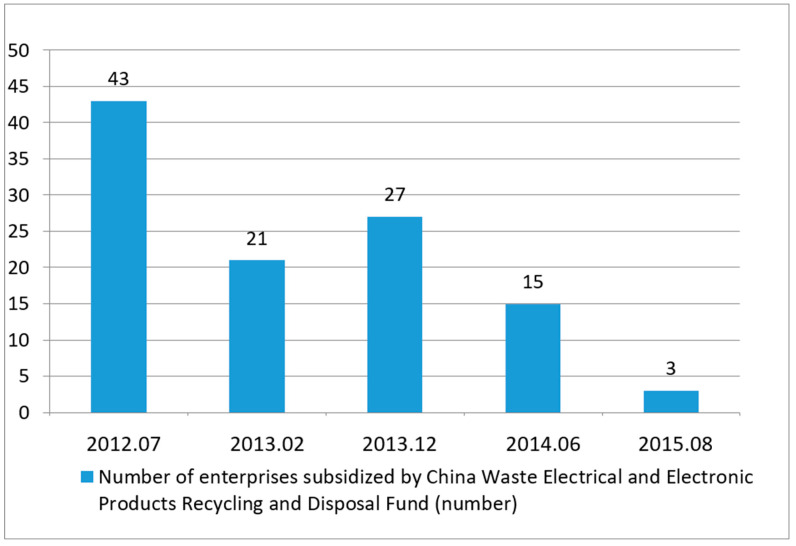
Number of enterprises subsidized by the recycling fund for WEEEs in China from 2012 to present.

By the end of November 2018, a total of 109 enterprises in five batches had entered the list of fund subsidies. The survey found that from 2013 to 2017, there were 48, 15 and 3 new companies in 2013, 2014, and 2015, respectively. No new companies entered the list in 2016 and 2017. The growth rate of the total number of enterprises entering the waste electronic dismantling industry has obviously slowed down and has been in a stagnant state in the past two years. The published number of WEEE rehabilitation fund subsidized enterprises in China is shown in Figure 8.

**Figure 9:**
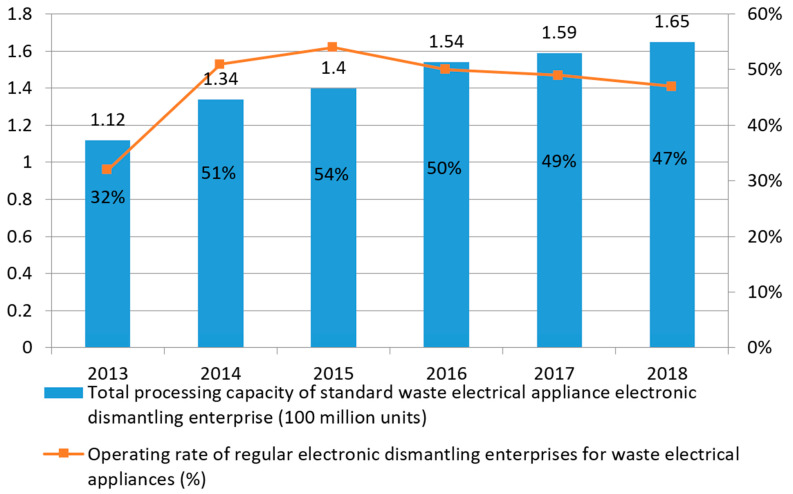
Capacity and operating rate of normal recycling and processing enterprises for WEEE in China (unit: 100 million units, %).

From 2013 to 2016, with the progress of dismantling technology and the development of industries, the electronic dismantling capacity of conventionally used electrical appliances in China increased from 112 million to 154 million, with a compound growth rate of 11.2%. However, the overall operating rate of the WEEE dismantling industry was only approximately 50%. In recent years, the operating rate has shown a downward trend, with an obvious idle capacity and a serious waste of capacity. The statistics of the Ministry of Environmental Protection of China are shown in Figure 9.

The industry is struggling. According to an investigative report on the operation status of 109 formal dismantling enterprises in China in 2018, one-third of the dismantling enterprises of WEEEs in China barely survived, nearly one-third faced bankruptcy, and the remaining one-third experienced a state of suspension of production.

There are many reasons for the low-capacity utilization rate in formal recycling enterprises. The labour requirements for recycling technology and equipment are relatively high, and enterprises need much investment and personnel training. Moreover, regular recycling as a system that does not have a stable commodity supply is unstable in construction, and enterprises still need out-of-stock costs and high recycling costs. Before 2016, the prices of iron, aluminia, plastics and other materials dismantled by WEEE showed a downward trend, which further reduced the profit level of enterprises and increased the operating pressure of enterprises.

Moreover, in terms of the competition between formal recycling companies and informal traders, the former is at a disadvantage in some respects. Formal recycling companies need to process products according to the requirements of national laws and regulations and must consider that the environmental impact is not directly proportional to their input and income. Informal suppliers resell discarded electronic products, such as mobile phones and other second-hand products, for huge profits, and the recycling price they provide is much higher than that of formal enterprises; thus, their market competitiveness is relatively low.

### 4.2. Issues in the Support of Dismantling Enterprises by Subsidies

Subsidies have been issued for a long time, and the state subsidy fund cannot meet its income. The system needs to be improved.

**Figure 10:**
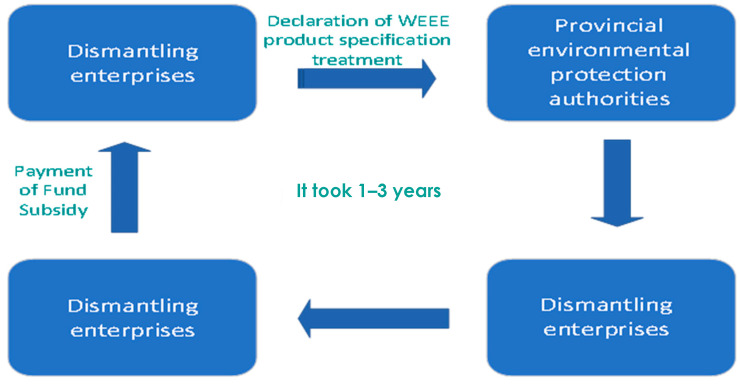
Schematic diagram of China’s EEE dismantling subsidy application process.

The application process for EEE dismantling subsidies in China is shown in Figure 10.

According to the data released in a white paper in 2017, the data on the collection and appropriation amount of WEEE funds from 2012 to 2017 are shown in Table 5:

The application for domestic financial subsidies is submitted to the provincial environmental protection department by the dismantling enterprise of WEEEs in the form of product variety and quantity reports. The provincial environmental protection department and the third party conduct written and onsite audits of the application and submit the audit results to the Ministry of Environmental Protection in the form of official documents (002E). After verification by the Ministry of Environmental Protection, the results are submitted to the Ministry of Finance, which will determine the amount of subsidies that each enterprise is entitled to and then distribute the subsidies. From the perspective of the subsidy payment process, the process involves provincial departments, the Ministry of Environmental Protection and the Ministry of Finance and has been reviewed many times.

The procedure is complicated, and the whole application process takes approximately 1–3 years at the earliest. Some enterprises did not even record dismantling subsidies in the second half of 2015. The failure to receive subsidy funds in time will directly lead to an increase in accounts receivable, a shortage of working capital and an increase in additional financial costs. For enterprises, although there is no need to worry about the risk of bad debts, capital cannot be recovered and the daily operation of enterprises is still under great pressure, especially for some smaller enterprises. When necessary, the dismantling plant must be compressed or even closed to ensure the normal turnover of the company.

The white paper on collecting the appropriate amount published by the government showed that manufacturers and importers have been managed since 2012 and the collection amount has reached a stable level since 2013, with fluctuations of approximately 2.8 billion yuan used for the treatment of used electronic and electrical products. The number of qualified enterprises has been rising, the processing technology of enterprises has gradually matured, the processing capacity has been continuously improved, and the total amount of government funds has increased rapidly. The balance of revenue and expenditure of national funds was negative from 2014 to 2016, and the balance of revenue and expenditure changed from negative to positive in 2017, which is mainly due to the government’s delay in granting subsidies. The shortage of funds is mainly because the producer’s payment standard is significantly lower than the subsidy standard. The difference in revenue and expenditure for each product is so large that the gap in total revenue and expenditure is obvious.

### 4.3. Issue with Improving the Formal Recycling Channel

The difficulties in improving the formal recycling channel lead to higher recycling costs for enterprises.

**Figure 11:**
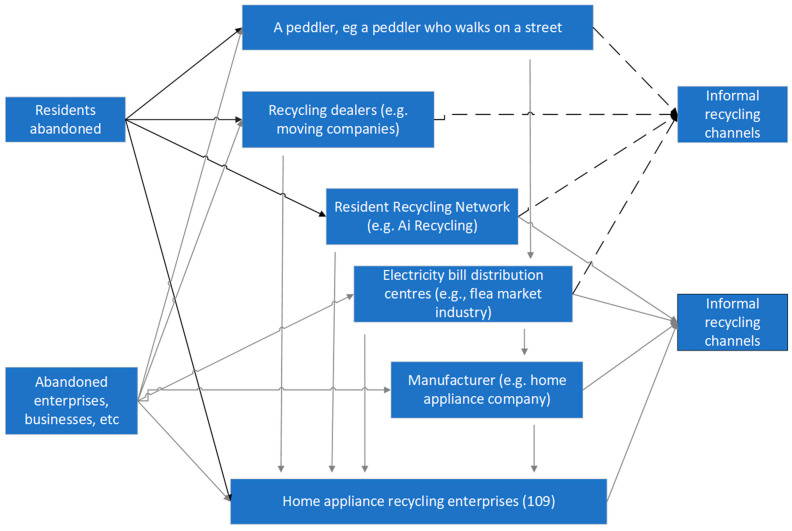
Channels of WEEE recovery in China.

Regular recycling enterprises are under great pressure to survive. The lag of government subsidies and the insufficient operating rate of enterprises have led to slow industry development. In addition, the construction of formal recycling channels in China is imperfect, which directly increases the recycling costs of enterprises and reduces the profit margins of enterprises. At present, the recycling channels of WEEEs from residents to dismantling enterprises in China are shown in Figure 11.

In China, WEEE recycling has developed into a situation in which street recycling, dealer recycling, community network recycling, distribution centre recycling, production enterprise recycling and WEEE recycling enterprise recycling coexist, thus forming an informal and formal dual-channel recycling system, and the competition pattern is even more chaotic. Due to the residents’ lack of understanding of the resources and pollution associated with waste electronic and electrical products and the lack of corresponding laws, regulations and standards for the discarding of waste electronic and electrical products by residents, the residents control the recycling channels to obtain higher prices or facilitate selling WEEE directly to the sellers. Thus, the situation among sellers will be difficult to change.

A total of 109 enterprises have obtained the national WEEE recycling qualification, and they perform recycling among residents, enterprises, businesses and other industries. In addition, they can also obtain waste power products through small vendors and other channels. However, the acquisition cost of such products will be higher and the difficulty and cost of dismantling and processing will be higher. Statistics show that by 2018, approximately 90% of the WEEE in China had not been standardized but was recycled by mobile vendors or directly recycled and disassembled by family dismantling workshops or small enterprises that had not obtained the national dismantling qualification.

Compared with developed countries, one of the important reasons for the difficulty in establishing formal recycling channels in China is that the scale of formal recycling enterprises lags behind informal channels. Statistics show that as of November 2018, there were more than 2000 qualified recycling enterprises of WEEEs in the United States, more than 500 in the European Union, and only 109 in China, which is far lower compared with that of developed countries.

In addition, China’s fund policy since 2014 also stipulates that when the actual annual dismantling capacity of various WEEEs is less than 20% of the enterprise’s licensed processing capacity, the fund subsidy qualification will be cancelled, which also makes many enterprises face enormous operating pressure.

## 5. Suggestions to Overcome the Challenges in Fund Policy Implementation in China

WEEE has become an important part of municipal waste, and it is also the fastest growing and most potentially dangerous waste in the world. However, WEEE also contains a large number of recyclable and renewable resources. Therefore, how the government decides to stimulate the effective recycling of WEEE is the focus of circular economic development in China.

GEM Co., Ltd. (GEM, Shenzhen, China) first proposed the concept of “exploring urban mines” and “limited resources, infinite recycling” in China. This company actively explores China’s mining mode of “urban mining” and is also committed to and leading in the research and industrialization of battery scrap, discarded electronic appliances, wasted rare metals and “urban mining” recycling and recycled products.

GEM initiated the Go Green recycling platform that represents an active practice in the development of green environmental protection, which is different from the traditional method of recycling. Go Green has introduced an innovative new business model “Internet +” (i.e., “Internet +” refers to the combination of internet and traditional industries. By leveraging the advantages of internet industries, development opportunities can be explored and created in traditional industries) in the recycling industry, and it offers convenience to both self-employed entrepreneurs and consumers. Go Green has also provided a number of recycling projects, including waste paper, waste plastics, electricity, and small appliances, among others.

### 5.1. Online to Offline (O2O) Classified Recycling Platform-Go Green

In 2015, the Third Session of the 12th National People’s Congress first proposed the “Internet +” action plan in the government work report and clearly pointed out in the “Circular Economy Promotion Plan 2015” that China will actively guide and promote the innovation of renewable resource recycling modes, explore new modes and paths, such as “internet plus” recycling, and support the development of new recycling methods, such as intelligent recycling and social automatic recycling machines.

O2O (online to offline) refers to the combination of offline business activities and the internet to make the internet a new platform for offline transactions. The most commonly used online platforms in China include Meituan and Didi Taxi, among others.

With the significant growth of the number of WEEEs, the hidden value inside the discarded products is significant. In this case, commercial activities regarding those products will inevitably appear. WEEE recycling is the complete “extraction” of resources and the pursuit of the highest utilization rate of original mineral resources; therefore, the concept of “urban mine” (UM) was proposed. Driven by government policies, China’s WEEE recycling and processing industry has moved towards a new development direction, and it has greatly promoted the popularization and participation of WEEE recycling and dismantling.

In the preliminary exploration of WEEE recycling in China, the traditional recycling method had little effect in practice; therefore, the first domestic O2O classified recycling platform, “Recycling”, was officially launched by the GME on 22 July 2015. The platform combines online and offline contexts, builds a “Go Green” website, and promotes and realizes business through mobile apps, official WeChat accounts and other channels.

The first batch of “Internet + Classified Recycling” urban waste classification and recycling systems in China was established. Through the operation of the platform, residents (WEEE source), Go Green platform users (recycling personnel), enterprises (production, dismantling and processing enterprises) and the government (policy support and incentives) were closely linked to realize the green life cycle, with a classified recycling system for WEEE initiated from abandoned sources.

The working principle of the “Go Green” platform is as follows: citizens need to register their personal accounts on the platform website or mobile app and include basic information, such as their address. When WEEE is produced, the users can communicate with the platform for processing or directly click on the online service requirements. At the appointed time, “Go Green” staff will arrive to collect the recycling items. In addition, the products can be evaluated on the spot, and the prices can be estimated. After the product is returned to the platform agent for professional evaluation, the evaluation results can be sent to the user. The amount of goods sold can be transferred to the user’s account on the same day.

### 5.2. Operation of the O2O Classified Recycling Platform

Taking “Go Green” as a reference, the mode of building an O2O-based WEEE recycling platform is described below.

The recycling process starts with the WEEE holders selling products, which reach the recycling site through the internet platform. The whole product (resource) information flow and capital flow are transmitted online, which is synchronized with the process of offline recyclers picking up goods, logistics transportation, recycling WEEE products with appropriate funds and selling WEEE products to recycling and processing enterprises.

Unlike the traditional O2O platform, the internet operation platform only needs to be responsible for online business, handle capital circulation, coordinate customer needs and protect the rights and interests of all parties. The recycling mode based on the O2O platform also takes offline business into account by requiring the establishment of physical service stores, such as agents and outlets. A certain number of professionals are required to pick up goods at home, identify the goods, store them temporarily and dispatch them to the corresponding dismantling and processing enterprises.

“Go Green” is known as the environmental protection service for classified recycling of WEEEs in this network age. The service teams are not unorganized, and they carry out internet recycling together with GEM. They are members of the company system with organized guarantees. After receiving professional training, they understand the norms and disciplines, and they are polite and have a good reputation. They have changed from street canvassers to green messengers to improve the urban environment, and they can receive orders online under recycling rules, perform unlimited business, and improve their quality of life through their own efforts. They are truly organized, disciplined and respected and represent multi-benefit environmental improvement and service teams in China, which can change the situation in which 90% of WEEEs in China are processed by informal recyclers. Moreover, the implementation of the platform can provide employment opportunities for a large number of people and alleviate their living difficulties.

For residents, WEEEs can be sold at home more conveniently and quickly; at the same time, the price of WEEEs is recycled in the system specification and professional on-site valuation of recycling is provided; therefore, there is no traditional channel for personnel bidding for recycling at random. Additionally, community activities can be held on “Resource Recovery Day” (launched in Shenzhen in June 2015 and will be held on Saturday), and residents’ participation is conducive to green consumption, green popularization and green recycling. Adults and children are called on to participate in activities and carry out “front-end” classification to solve the WEEE classification problem from the source. On the recycling day, “high-value WEEE can be exchanged for cash, and low-value WEEE can be exchanged for points and prizes”. These activities not only improve the recovery rate of WEEEs but also improve the participation of residents.

Recycling operators on the internet, on the one hand, only need to provide funds and recycle WEEEs from regular recyclers at the purchase price. In the fast cycle, capital funds for system specifications can clarify the surplus value of a product and accumulate product classifications. With such operations, both consumers and recycling enterprises have benefited greatly. On the other hand, they have to compete with other informal recycling personnel together; therefore, their industry competition is clearer, the job is more secure, unnecessary fierce competition is avoided, and the cost of enterprises is reduced, and these factors are conducive to the development of the industry.

This “Internet +” O2O classified recycling platform model can standardize formal recycling channels, reduce the coverage of informal recyclers, classify WEEE front-ends, and greatly reduce the recycling cost of processing enterprises. Meanwhile, WEEE has a high recycling rate and a perfect recycling system; therefore, manufacturers can track their own product flow and provide ideas for their own product design. While improving the start-up of recycling enterprises, recycling enterprises can also process products according to their own product categories and scales, which can improve their processing capacity and reduce processing costs, help address the development of the industry, improve the profitability of the industry, effectively alleviate the delay of government subsidies and reduce the pressure on enterprises. Moreover, the complete resource database and information management system of the internet platform can provide insights for the government for determining pricing and recycling subsidies for manufacturers’ products.

### 5.3. Development Limitations and Suggestions for the “Internet +” Recycling Model

Regarding the development limitations, first, the “Internet +” recycling model requires a high degree of specialization for internet recycling operators, namely, third-party payment platforms. In the whole recycling process, the internet platform needs to be responsible for all online business, including the daily updating of the platform, and it also needs to take into account offline recycling network construction and the WEEE circulation warehousing business, which plays an irreplaceable role in the whole recycling system. Moreover, there are many kinds of WEEE resources, and the early recycling platform structure of the internet has higher technical requirements for WEEE classification, testing and evaluation, which need to be addressed by internet recycling operators. Therefore, we need a large number of experts and a large number of advanced technologies and equipment. The “Internet +” recycling model is not sustainable. However, China’s waste electronic and electrical product recycling technology and network platform construction are still not perfect and remain in the exploratory stage; thus, future development will face many challenges.

Second, residents may still not grasp the concepts of environmental protection and green recycling, and the recycling network platform has not yet been popularized. There are many kinds and large quantities of WEEE, and the traditional recycling mode has a large control range. When the “Internet +” recycling mode and the traditional recycling mode begin to collide, the internet platform will inevitably be at a disadvantage. Under the traditional recycling mode, many small traders will choose illegal recycling, which will lead to unfriendly behaviours, such as environmental pollution. The trends of interest include environmental pollution and a lack of knowledge. This widespread illegal behaviour is still difficult to monitor, which will hinder the development of innovative recycling modes.

**Figure 12:**
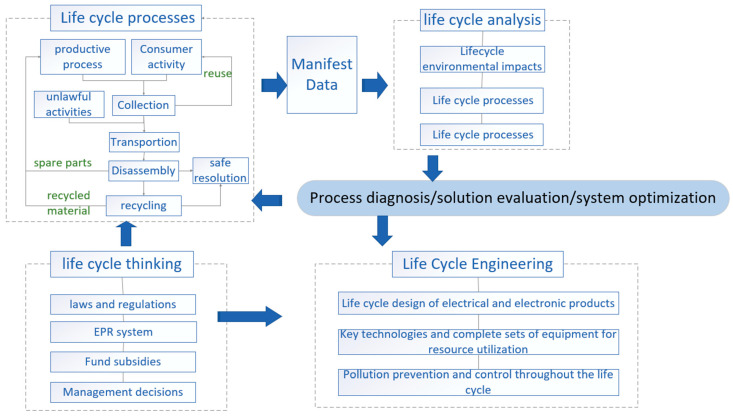
WEEE lifecycle management system framework.

According to research on the life cycle assessment method and its application in WEEE, the management system framework for product-based life cycle assessments can be expressed as shown in Figure 12:

Since the emergence of WEEE, life cycle assessment analyses have been performed to identify different life cycle processes of e-waste by introducing life cycle ideas (including laws and regulations, systems, financial subsidies, and government management), conducted life cycle analysis (including environmental impact, cost and social impact) through quantitative and qualitative methods, and then implemented life cycle engineering via evaluation and data analysis, technology optimization, and product design innovation. In the whole life cycle management, such analyses focus on the whole process from waste generation to final treatment and adopt a series of theories and engineering technologies, such as green design, efficient recycling and dismantling, resource treatment, and product design, production, use and scrap recycling.

The establishment of an “Internet +” recycling platform is conducive to the establishment of formal recycling channels. The WEEE fund policy of the government only subsidizes formal processing enterprises. The O2O recycling platform classifies WEEE sources, which not only reduces the pressure of recycling enterprises but also greatly promotes the sustainable development of society. On the one hand, the government needs incentives and certain rewards, and the internet platform, which is at the beginning of platform construction, requires sufficient funding and must introduce a large number of professionals. The government needs to help enterprises build platforms, reduce costs, and support the popularization of the operation and development of the “Internet +” recycling concept. In the implementation of capital policy, the recycling platform can be considered the subsidy object.

## 6. Conclusions

Since the implementation of the funding policy in 2012, China’s waste EEE industry has made great progress, which is of great significance to the sustainable development of human societies. The implementation status of a funding policy is complicated, and many scholars have conducted in-depth research and discussed various aspects of funding policy. Since Internet + recycling was proposed in 2015, new development directions have been considered for the WEEE recycling industry.

This paper makes an important theoretical contribution to the WEEE management and circular economy literature by analyzing the concepts, characteristics and resource circulation channels of the circular economy, which is an EPR, and the life cycle evaluation method. Also, through the case of GEM, the implementation of a circular economy and EPR is well demonstrated. Additionally, the paper promotes a better understanding of the development origins and their positive impact on society and the synchronous and mutual development of WEEE recycling by the promotion and implementation of WEEE fund policies.

Additionally, this paper has managerial implications for WEEE management and WEEE fund management. This paper studied the operation mode of WEEE funds in various developed countries and regions and compared their operation modes with that in China. The operation mode of the WEEE fund in China is similar to that in foreign countries, although space to learn and improve is observed. Certain gaps in efficiency and cost are observed relative to that in foreign developed countries and regions, and problems in the recycling mode of developed countries and regions are also observed. Practice has shown that directly learning from foreign fund operation modes is infeasible; thus, it is particularly important to constantly improve and innovate the WEEE fund operation mode that conforms to China’s national conditions. In addition, this paper proposed the O2O recycling platform based on the “Internet +” recycling mode, which can effectively solve the problems encountered by WEEE recycling in China and provide a reference for China’s WEEE fund policy improvement.

However, this paper is a desk-based study, and it is expected to see more case studies to justify the feasibility of the mode of a circular economy in WEEE management. Also, the mode of circular economy is one feasible mode of WEEE management, but with the rapid speed of technology iteration nowadays, it is expected that there will be more innovative approaches for WEEE management. Additionally, this paper concluded the “Internet +” recycling mode can solve the problems of WEEE recycling in China. However, whether such a mode can be directly applied or modified in other countries, especially developing countries, deserves further consideration. Moreover, China reviewed the WEEE fund in March 2021, and the future research on China’s WEEE funding policy can be based on the new regulation, which may bring about new insights into China’s WEEE management and fund operation mode.

## Figures and Tables

**Table 1:** Comparative analysis of the different fund operation characteristics.

	Fund Collection Mode	Fund Subsidy Beneficiary	Capital Operation Agents	WEEE Policy Management
Germany	Producer payment	Collectors	PRO	Government
Japan	Customer payment	Dismantling companies	National institution	Customers and manufacturers
The Netherlands	Producer payment	Collectors and dismantling companies	PRO	Government
China	Producer payment	Dismantling companies	National institution	Government

**Table 2:** Comparison between the old and new WEEE fund subsidy standards in 2016.

	Product Name	The Old Standard θ1 (Yuan/Units)	New Standard (Yuan/Units) θ2		
			Model		Remarks
1	Television	85	Cathode-ray tube (black and white, color) TV set of 14 “and above and 25” and below	60 (down 25)	Cathode-ray tube (black and white, color) TV sets under 14 inches will not be subsidized
			Cathode-ray tube (black and white, color) TV set, plasma TV set, LCD TV set, OLED TV set, rear projection TV set	70 (down 15)	
2	Washing machines	35	Single bucket washing machine, dehydrator (3 kg < dry clothes capacity ≤ 10 kg)	35	Washing machines with dry volume ≤3 kg will not be subsidized
			Double drum washing machine, wave wheel type automatic washing machine, drum type automatic washing machine (3 kg < dry quantity ≤ 10 kg)	45 (Raised 10)	
3	Refrigerator	80	Refrigeration Refrigeration (cabinet), Refrigeration (cabinet), Refrigeration (cabinet) (50 L ≤ volume ≤ 500 L)	80	Refrigerators with capacity less than 50 litres will not be subsidized
4	Microcomputer	85	Desktop microcomputer (including host computer and monitor), desktop microcomputer in the form of host monitors integrated, portable microcomputer.	70 (down 15)	Standards for tablet computers and mobile phones will be formulated separately
5	Room air conditioners	35	Integral air conditioners, split air conditioners, multi-towed air conditioners (including outdoor and indoor units) (refrigerating capacity ≤14,000 W)	130 (Raised 95)	

**Table 3:** Scope and standards of the funds levied by China on enterprises selling EEEs.

	Product Type	Product Scope	Collection Standard (Yuan/Set)
1	Television	Cathode-ray tube (black and white, color) TV sets, liquid crystal TV sets, plasma TV sets, back projection TV sets, other terminal equipment used for receiving signals and restoring images and audio	13
2	Washing machines	Wheel washing machines, drum washing machines, agitator washing machines, dehydrators, and other appliances for washing clothes by mechanical action (including both dry-clothes functions)	7
3	Refrigerator	Refrigerated freezers (cabinets), freezers (cabinets), freezers (cabinets), other insulated boxes having refrigeration systems and consuming energy to obtain cooling capacity	12
4	Microcomputer	Desktop microcomputer monitor, host computer, monitor integrated form of desktop microcomputer, portable microcomputer (including tablet computer, palm computer), other information transaction processing entities	10
5	Room air conditioners	Integral air conditioners (window machines, wall machines, etc.), split air conditioners (split wall hangers, split cabinet machines, etc.), multi-air conditioners, and other room air conditioners with cooling capacity of 14,000 W and below	7

**Table 4:** Changes in retention, scrap and disassembly in 2016/2017.

Product Type	Subsidized Price(θ2/θ1)	Ratio of Household Ownership (2017/2016)	From 2016 to 2017, Theoretical Scrap Increased Year on Year		Ratio of Actual Disassembly Volume (2017/2016)
			Quantity	Weight	
Television 1	0.706	1.006	0.051	0.050	0.921
Television 2	0.824				
Washing machines 1	1.000	1.042	0.104	0.101	0.935
Washing machines 2	1.286				
Refrigerator	1.000	1.049	0.139	0.140	1.109
Microcomputer	0.824	1.000	0.155	0.155	0.921
Room air conditioners	3.714	0.999	0.155	0.155	2.562

**Table 5:** Government fund policy collection, appropriation amount, and balance of income and expenditures.

	2012	2013	2014	2015	2016	2017
Amount collected (100 million yuan)	8.5	28.1	28.8	27.2	26.1	28.1
Amount allocated ($100 million)	0	7.5	33.9	54.0	47.1	0.7
Balance of revenue and expenditures (100 million yuan)	8.5	20.6	−5.1	−26.8	−27	27.4

## Data Availability

The data that support the findings of this study are available on request from the corresponding author. The data are not publicly available due to privacy or ethical restrictions.
